# Iron-Rim Lesions in the Optic Chiasm in Multiple Sclerosis: A Neuropathologic Case Series

**DOI:** 10.1212/WNL.0000000000214515

**Published:** 2026-01-22

**Authors:** Andrew Lockhart, Marco Pisa, Hal Drakesmith, Gabriele C. DeLuca

**Affiliations:** 1 Nuffield Department of Clinical Neurosciences, University of Oxford; United Kingdom; and; 2 MRC Weatherall Institute of Molecular Medicine, University of Oxford, United Kingdom.

## Abstract

**Objectives:**

The aim of this study was to demonstrate the presence of iron-rim lesions (IRLs) in the anterior visual pathway in multiple sclerosis (MS).

**Methods:**

Postmortem formalin-fixed paraffin-embedded anterior visual pathway tissues (optic chiasms) from 3 donors with MS were examined for iron using Turnbull Blue histochemistry. Demyelinated lesions were identified and staged with immunohistochemistry for myelin proteolipid protein and CD68.

**Results:**

A total of 9 lesions were identified, all of which were mixed active/inactive. Iron-positive microglia and macrophages were identified at the inflammatory borders in all 3 cases, in 8 of the 9 lesions. Within the inactive core, iron-positive astrocytes and, rarely axons, could be seen. Iron-positive macrophages were also seen in nonlesional white matter.

**Discussion:**

IRLs are common in mixed active/inactive MS lesions in the anterior visual pathway. IRLs are the pathologic correlate of paramagnetic rim lesions (PRLs) on MRI, the number of which in the brain correlates with the extent of disability. PRLs have, heretofore, not been described in the anterior visual pathway. Future work aimed at overcoming the challenges in detecting these lesions in the anterior visual pathway is warranted, to ensure that extent of chronic inflammation is not underestimated.

## Introduction

Multiple sclerosis (MS) is a chronic inflammatory demyelinating and neurodegenerative disease of the CNS. Involvement of the anterior visual pathway in MS is common at onset and throughout the disease course.^1^ Pathologic studies highlight that demyelinated lesions, including mixed active/inactive ones, are common in the anterior visual pathway in progressive MS,^2^ and optic nerve lesions relate to progressive optic atrophy.^1^

Some mixed active/inactive lesions in the MS brain demonstrate iron-laden microglia/macrophages along their rims and constitute the substrate of the paramagnetic rim lesion (PRL), visible on susceptibility-based imaging in vivo.^3^ PRLs are useful as a diagnostic and prognostic biomarker. Recently revised diagnostic criteria include the presence of PRLs as an indicator of disease specificity.^4^ The number of PRLs correlates with the extent of disability and predicts progression independent of relapse activity (PIRA).^5^ However, neither iron-rim lesions (IRLs) nor PRLs have been reported in the anterior visual pathway in MS, highlighting the need for further pathologic characterization of this clinically eloquent region.

In this case series, we herein report the presence of mixed active/inactive lesions containing iron rims along their borders.

## Methods

Ten pathologically confirmed archival MS cases with anterior visual pathway tissue (optic chiasm) from the Oxford Brain Bank were selected for study. These cases were selected from a previously published cohort^2^ (donations dated back to the 1970s, with limited clinical information). Formalin-fixed paraffin-embedded tissue was used to immunolabel for myelin proteolipid protein (PLP) and microglia/macrophages (CD68) to screen for the presence and stages of lesions using established criteria.^6^ Two observers (A.L. and M.P.) assessed lesions for the presence of IRLs. IRLs were defined by the presence of iron-positive microglia/macrophages at the CD68^+^ macrophage-rich lesional rim, in line with established guidelines for PRLs.^3^

Total nonheme iron was visualized using diaminobenzidine (DAB)-enhanced Turnbull Blue (Tiermann-Schmertzer method) staining, as previously described.^7^ After deparaffinization and rehydration, sections were incubated in 2% ammonium sulfide before treatment with 10% potassium ferricyanide and 2% hydrochloric acid for 30 minutes. Endogenous peroxidase activity was quenched with 0.3% hydrogen peroxide in methanol. Sections were rinsed with phosphate buffer solution (pH 7.4) before being developed in DAB. Hematoxylin was used as a counterstain.

### Standard Protocol Approvals, Registrations, and Patient Consents

Tissue was obtained from the Oxford Brain Bank with appropriate research ethics committee approval (REC 15/SC/0639).

### Data Availability

Raw data are available on appropriate request to the corresponding author.

## Results

Of the 10 cases with optic chiasms available, only 3 cases were amenable to study. Prolonged formalin fixation times in 6 cases (>20 years in 4 cases) rendered them unsuitable for Turnbull histochemistry, with no true staining. One case was entirely nonlesional. The Table lists the patient characteristics of the studied cohort.

**Table:** Demographic Characteristics of Patients

Case	Disease duration (y)	Clinical phenotype at death	Documented optic neuritis	Postmortem interval (hrs)
MS1	30	SPMS, noted to have an intrathecal baclofen pump	Unknown	39
MS2	20	SPMS, significant disability, with paraplegia and significant ataxia	Yes, severe bilateral ON several years into illness, with some recovery	33
MS3	20	Unknown	Unknown	26

Abbreviations: SPMS=Secondary Progressive Multiple Sclerosis; ON=Optic Neuritis.

Two donors were female and 1 male (not included in the Table for anonymization). Age range: 45–74 (individual ages not included for anonymization).

**Figure 1:**
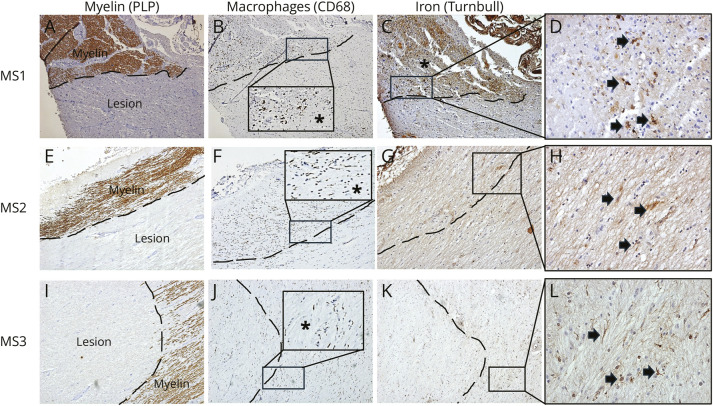
Iron-Rim Lesions Are Present in the Anterior Visual Pathway in MS Anterior visual pathway sections from 3 MS cases are shown (MS1, 2, and 3). (A, E, I) Demyelinated lesions using myelin proteolipid protein (PLP) immunohistochemistry; myelinated areas appear brown. (B, F, J) Inflammatory microglia/macrophages at the lesion edge with CD68 immunohistochemistry, confirm that these lesions are mixed active/inactive. Note in each inset prominent microglia/macrophages at the rim and fewer microglia/macrophages in the lesion core (marked by *). Note that the inflammatory edge is not prominent in MS3. (C, D, E, H, K, L) Iron-containing microglia/macrophages at the lesional edge using modified Turnbull Blue histochemistry (black arrows highlight the iron + microglia/macrophages, brown color), confirming that these lesions are IRLs. Note the diffuse DAB positivity in the nonlesional tissue in MS1 (marked by *), likely representing iron stores within intact myelin. Magnification for A–C, E–G, and I–K is ×40 and for D, H, and L is ×400.

**Figure 2:**
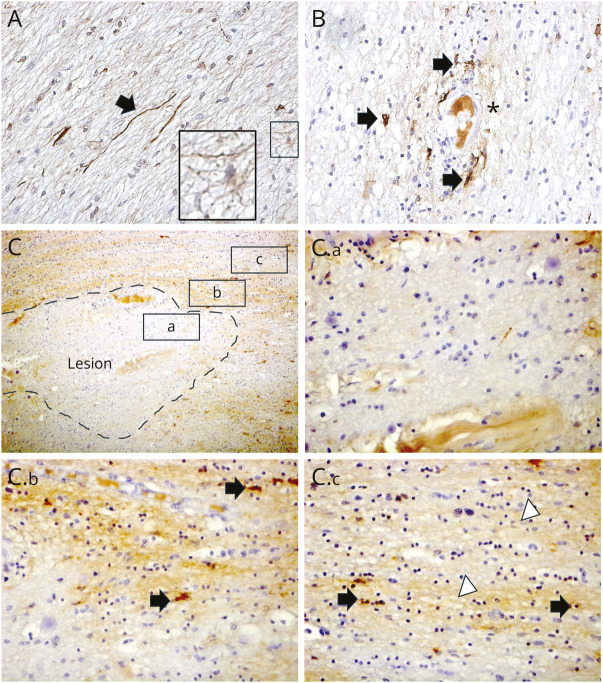
Additional Features of Total Nonheme Iron Histochemistry in the Anterior Visual Pathway in MS (A) Within some inactive lesion cores, iron-positive astrocytes (highlighted in the inset) and iron-positive axons (black arrows) could be seen. (B) In nonlesional white matter, iron-positive microglia/macrophages were seen (black arrows), especially around blood vessels (marked with *). (C and C.a, C.b, C.c) Iron reactivity in IRLs. An example of an IRL from MS1 is shown (C), highlighting the lesion core (a), rim (b), and adjacent nonlesional white matter (c). In the lesion core (C.a), there was loss of iron reactivity. At the rim (C.b), iron-positive microglia/macrophages were seen (black arrows). In adjacent nonlesional white matter (C.c), iron-reactive glia with an oligodendrocyte morphology could be seen (black arrows) and there was diffuse iron reactivity, with a “fluffy” appearance, consistent with myelin (white arrowhead). All of these features have been reported in IRLs in the brain (Ref. 8). Magnification is at ×400, except C, which is at ×100.

A total of 9 lesions were identified across the 3 cases, all of which were mixed active/inactive. Iron-positive microglia/macrophages were visible at the inflammatory edge in 8 of the 9 lesions (examples shown in Figure 1). The number of iron-positive microglia/macrophages within the IRLs was modest in comparison with the extent of CD68^+^ cellular infiltration, except in 1 case (MS1, Figure 1) where iron-labeled macrophages at the rim were abundant. Within the inactive lesional core, iron-positive astrocytes and, rarely axons, could be seen (Figure 2A). Lesional cores exhibited reduced iron reactivity in the 8 IRLs, as previously described^3,7^ (Figure 2C, C.a, C.b, C.c).

In nonlesional white matter, iron was predominantly found in glial cells resembling oligodendrocytes, within the myelin, and less frequently in microglia/macrophages, particularly around blood vessels (Figure 2B).

## Discussion

In pathologic studies, the presence of mixed active/inactive lesions is commonplace in the anterior visual pathway, as is seen in other nervous system areas at end-stage disease.^2^ Retinal atrophy measured by Optical Coherence Tomography (OCT) correlates with disability accumulation and PIRA.^9^ We extend these findings by showing that a large proportion of mixed active/inactive lesions in the anterior visual pathway have iron-rim borders, consistent with IRLs. In addition, we noted some iron-positive microglia/macrophages in nonlesional tissue, which has been reported previously in the brain.^7^ The significance of this is not yet known but may suggest more widespread iron dysmetabolism in MS.

IRLs are the pathologic correlate of PRLs, which can be detected in vivo with susceptibility-based MRI. PRLs in the anterior visual pathway have not been reported in MS, likely because of considerable challenges of optic nerve imaging including the small size of the optic nerve, and CSF and motion artifacts.^1^ Our pathologic findings would predict the presence of PRLs in the anterior visual pathway, highlighting a need to navigate these technical challenges and detect PRLs, which are of diagnostic and prognostic value.^5^ Advances in real-time motion correction, image reconstruction, and improvements in susceptibility-based imaging may assist in characterization of optic pathway lesions.

PRLs are a promising predictive biomarker of disability in MS. A larger number of PRLs in the brain correlate with worse disability scores, brain and spinal cord atrophy, higher levels of neurofilament light chain, and retinal thinning on OCT^10^ and are predictive of PIRA events^5^ as recently reviewed.^3^

Tracking neurodegenerative changes along the optic pathway through OCT has proven to be a sensitive biomarker for MS pathology,^11^ supporting the importance of developing other biomarkers of optic nerve pathology such as PRLs. Moreover, optic nerve lesions correlate with progressive optic atrophy,^9^ and PRLs are associated with more destructive lesion pathology.^3^ It is not known whether PRLs in the anterior visual pathway predict progressive retinal thinning. Although this study cannot address this, such an association warrants study, given the more destructive nature of these lesions in the brain.

MS lesions typically display a predilection for certain regions of the CNS, including optic nerves, periventricular white matter, and cervicothoracic spinal cord. Despite this, the topographical distribution of PRLs is not well described, with many articles reporting PRL number and volume, but not location. Some articles have reported topographical variation among periventricular, juxtacortical, and cortical lesions (e.g., 8, summarized in Ref. 3), as well as their relationship to the vasculature.^12^ Only 1 study from China has systematically reported on the topographical variation in PRLs, with periventricular and juxtacortical areas having highest prevalence of PRLs, and cortical and infratentorial PRLs being less common.^13^

Mixed active/inactive lesions are prevalent in several CNS areas at end-stage disease, including the anterior visual pathway.^2^ On the contrary, PRLs account for only approximately 10% of MS lesions, suggesting that some mixed active/inactive lesions do not have detectable iron at the lesional edge.^6^ The variability in detection of PRLs throughout the CNS may be due to several factors including differences in regional myelin iron content^14,15^ and imaging technical limitations.^1^ The use of number of PRLs as a prognostic marker needs to consider these topographical nuances. PRL formation may depend on both the amount of iron within the myelin of a given CNS region and the number of inflammatory macrophages at the lesional rim. If people with MS are to be stratified based on the number of PRLs, it is important that PRLs in all regions of the CNS are considered, so that pathology in certain topographies is not under-represented.

In summary, we report the presence of IRLs in the anterior visual pathway in a pathologic cohort of progressive MS donors. The main limitation of this pathologic case series is that it is restricted to postmortem analysis of a small number of cases, in whom clinical details are limited. All 3 patients had longstanding disease, so we cannot comment on IRL presence in early MS. Future work evaluating the pathologic extent, distribution, and clinical correlation of IRLs in the anterior visual pathway in larger postmortem cohorts and their detection in vivo will assist in the appropriate inclusion of PRLs in clinical practice.

## References

[1] Sastre-GarrigaVidal-Jordana JA, Pareto D, Rovira À, et al. Value of optic nerve MRI in multiple sclerosis clinical management: a MAGNIMS position paper and future perspectives. Neurology. 2024;103(3):e209677. doi:10.1212/WNL.0000000000209677 39018513 PMC11271394

[2] Pisa M, Pansieri J, Yee S, et al. Anterior optic pathway pathology in CNS demyelinating diseases. Brain. 2022;145(12):4308-4319. doi:10.1093/brain/awac030 35134111 PMC9762948

[3] Bagnato F, Sati P, Hemond CC, et al. Imaging chronic active lesions in multiple sclerosis: a consensus statement. Brain. 2024;147(9):2913-2933. doi:10.1093/brain/awae013 38226694 PMC11370808

[4] Montalban X, Lebrun-Frénay C, Oh J, et al. Diagnosis of multiple sclerosis: 2024 revisions of the McDonald criteria. Lancet Neurol. 2025;24(10):850-865. doi:10.1016/S1474-4422(25)00270-4 40975101

[5] Reeves JA, Mohebbi M, Wicks T, et al. Paramagnetic rim lesions predict greater long-term relapse rates and clinical progression over 10 years. Mult Scler. 2024:13524585241229956. 10.1177/13524585241229956 PMC11009059 38366920

[6] Dal-Bianco A, Oh J, Sati P, Absinta M. Chronic active lesions in multiple sclerosis: classification, terminology, and clinical significance. Ther Adv Neurol Disord. 2024;17:17562864241306684. doi:10.1177/17562864241306684 39711984 PMC11660293

[7] Hametner S, Wimmer I, Haider L, Pfeifenbring S, Brück W, Lassmann H. Iron and neurodegeneration in the multiple sclerosis brain. Ann Neurol. 2013;74(6):848-861. doi:10.1002/ana.23974 23868451 PMC4223935

[8] Jang J, Nam Y, Choi Y, et al. Paramagnetic rims in multiple sclerosis and neuromyelitis optica spectrum disorder: a quantitative susceptibility mapping study with 3-T MRI. J Clin Neurol. 2020;16(4):562-572. doi:10.3988/jcn.2020.16.4.562 33029961 PMC7542003

[9] Bsteh G, Hegen H, Altmann P, et al. Retinal layer thinning is reflecting disability progression independent of relapse activity in multiple sclerosis. Mult Scler J Exp Transl Clin. 2020;6(4):2055217320966344. doi:10.1177/2055217320966344 33194221 PMC7604994

[10] Krajnc N, Hofer L, Föttinger F, et al. Paramagnetic rim lesions are associated with inner retinal layer thinning and progression independent of relapse activity in multiple sclerosis. Eur J Neurol. 2025;32(1):e16529. doi:10.1111/ene.16529 39529542 PMC11622274

[11] Pisa M, Croese T, Dalla Costa G, et al. Subclinical anterior optic pathway involvement in early multiple sclerosis and clinically isolated syndromes. Brain. 2021;144(3):848-862. doi:10.1093/brain/awaa458 33829250

[12] Toubasi AA, Eisma JJ, Wang J, et al. Chronic active lesions preferentially localize in watershed territories in multiple sclerosis. Ann Clin Transl Neurol. 2024;11(11):2912-2922. doi:10.1002/acn3.52202 39447194 PMC11572742

[13] Liu X, Wang Y, Wei N, et al. The characteristics and influencing factors of paramagnetic rim lesions in Chinese MS patients: a 7T MRI study. Mult Scler J. 2025;0(0):13524585251328902. 10.1177/13524585251328902 40219829

[14] Bagnato F, Hametner S, Yao B, et al. Tracking iron in multiple sclerosis: a combined imaging and histopathological study at 7 tesla. Brain. 2011;134(Pt 12):3602-3615. doi:10.1093/brain/awr278 22171355 PMC3235560

[15] Morris CM, Candy JM, Oakley AE, Bloxham CA, Edwardson JA. Histochemical distribution of non-haem iron in the human brain. Acta Anat (Basel). 1992;144(3):235-257. doi:10.1159/000147312 1529678

